# Electrically Active Defects and Traps and Their Relation to Stoichiometry and Chemical Environment in HfO_2_/Al_2_O_3_ Dielectric Stacks as Revealed by XPS

**DOI:** 10.3390/ma18235420

**Published:** 2025-12-01

**Authors:** Dencho Spassov, Albena Paskaleva, Ivalina Avramova, Wojciech Wozniak, Elzbieta Guziewicz

**Affiliations:** 1 Institute of Solid State Physics, Bulgarian Academy of Sciences, 72 Tsarigradsko Chaussee, 1784 Sofia, Bulgaria; d.spassov@issp.bas.bg; 2 National Centre of Excellence Mechatronics and Clean Technologies, Kl. Ohridski Blvd, 8, Bl. 8, BG-1000 14, 1756 Sofia, Bulgaria; 3 Institute of General and Inorganic Chemistry, Bulgarian Academy of Sciences, 1113 Sofia, Bulgaria; iva@svr.igic.bas.bg; 4 Institute of Physics, Polish Academy of Sciences, Al. Lotników 32/46, 02-668 Warsaw, Poland; wwozniak@ifpan.edu.pl (W.W.); guzel@ifpan.edu.pl (E.G.)

**Keywords:** non-volatile flash memory, charge trapping, atomic layer deposition (ALD), HfO_2_/Al_2_O_3_ stacks, Al-doped HfO_2_, X-ray photoelectron spectroscopy (XPS)

## Abstract

Charge-trapping memory (CTM) is a viable contender to supersede the floating gate technology in high-density flash memory applications. To this end, very reliable charge storage in CTM should be secured. This requires optimization of trap density, their energy and spatial location as well as a deep understanding of their origin. In this work, we used X-ray photoelectron spectroscopy (XPS) to investigate chemical bonds in nanolaminated and doped HfO_2_/Al_2_O_3_ stacks in an effort to gain insight into the nature of defects in the electron/hole trapping processes. The impact of Al incorporation into the HfO_2_ and rapid thermal annealing (RTA) in O_2_ on the composition, stoichiometry and bonding configurations was studied. Incorporation of Al into HfO_2_ leads to an increased concentration of Hf-suboxides. Subsequent RTA effectively reduces suboxides, enhances the stoichiometry of the HfO_2_/Al_2_O_3_ stacks and facilitates intermixing at the dielectric interface, resulting in the formation of Hf–Al–O bonds. The valence band spectra indicate that both Al incorporation and RTA change the dielectric/Si band alignment in a similar way, lowering the valence band offset. The observed changes were considered in relation to the electrically active defects and traps in the structures.

## 1. Introduction

In recent years, flash memory has emerged as the fastest-growing segment of semiconductor products. To meet the demands of advanced ultra-high-density flash memory architectures, such as 3D-NAND, there is a growing need to replace the conventionally used silicon nitride (Si_3_N_4_) with high-k dielectric materials in the current charge-trapping memory (CTM) [1,2,3]. This transition aims to reduce the overall effective oxide thickness of the memory stack, thereby enhancing device performance. Notably, high-k materials have relatively high dielectric constant and large conduction band offsets with Si and tunnel oxide and are known for their trap-rich electronic structure—a characteristic typically considered detrimental in other nanoelectronic applications, such as logic devices [4,5,6]. In the context of CTM, however, the traps can be highly beneficial. A number of requirements for charge traps must be met to fully realize the potential of CTM technology. Specifically, their density, energy levels and spatial distribution should be carefully optimized to minimize leakage and variability, prevent thermal de-trapping and reduce the risk of lateral charge spreading. Moreover, these traps must maintain their characteristics at high temperatures, for long retention times and under cycling stress while exhibiting fast charge capture and emission [7]. Meeting these criteria ensures efficient and reliable charge trapping and storage, as well as fast programming and erasing—key factors for the enhanced performance and endurance of CTM devices. Achieving this requires a deep understanding of the origin of the traps, their energy and spatial distribution and the strategies available to tailor their properties. Viable approaches to modify the trap properties include doping/mixing of high-k dielectrics with other elements, annealing steps, UV irradiation, bandgap engineering, etc. [8,9,10].

Among the promising high-k dielectrics considered as a possible charge-trapping layer in CTM, HfO_2_-based high-k dielectrics fabricated using atomic layer deposition (ALD) stand out due to the substantial knowledge acquired on their properties and their well-established integration in advanced microelectronic applications. In particular, introducing Al in HfO_2_ or stacking HfO_2_ with Al_2_O_3_ has been reported to improve the charge-trapping ability and enhance the memory performance and reliability of CTM [11,12,13,14]. In our previous studies [15,16,17,18], we have demonstrated that charge-trapping layers (CTLs) incorporating HfO_2_/Al_2_O_3_ stacks exhibit superior charge-trapping efficiency compared to Si_3_N_4_. Moreover, the charge-trapping performance of HfO_2_/Al_2_O_3_ CTLs can be tailored and enhanced through optimization of the stack parameters and annealing processes. In particular, we have investigated CTLs with various Al-layer thickness and number of HfO_2_/Al_2_O_3_ interfaces subjected to RTA in O_2_ or N_2_. The results revealed that the annealing in O_2_ affects stronger the charge-trapping ability of the stacks while the existence of HfO_2_/Al_2_O_3_ interfaces influences it only slightly with nanolaminated stacks demonstrating stronger electron trapping than the doped ones; neither the retention of electrons nor their discharge mechanism or discharge rate depend on the CTL [17]. These results imply that the same type of trap is responsible for electron trapping in both types of CTL, which gives us a reason to conclude that the traps are related to Al_2_O_3_ rather than to the number of interfaces. Aluminum introduces deep, high-density traps without increasing leakage currents, while O_2_ annealing further enhances electron trapping in HfO_2_/Al_2_O_3_ stacks. Consequently, the combination of Al incorporation into HfO_2_ and O_2_ annealing yields improved trapping and storage capabilities, reflected in large memory windows as well as excellent retention and endurance of the CT stacks. However, the origin of this improvement is unclear and remains a subject of ongoing investigation.

In this work, the stoichiometry and chemical environment of hafnium in HfO_2_/Al_2_O_3_ stacks and their band alignment with Si were studied by XPS and correlated to oxide charges and traps in the stacks.

## 2. Materials and Methods

**Figure 1:**
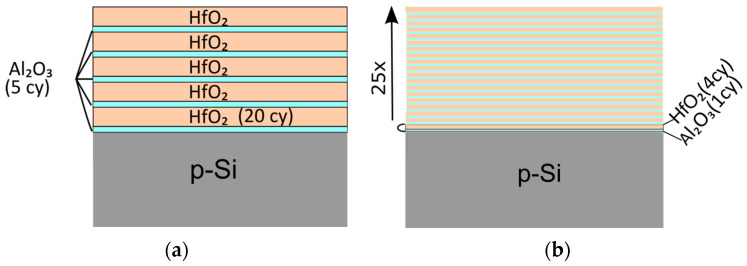
Schematic presentation of nanolaminated (**a**) and doped (**b**) HfO_2_/Al_2_O_3_ stacks.

Two types of HfO_2_-Al_2_O_3_ stacks were prepared by ALD: (i) nanolaminated HfO_2_/Al_2_O_3_ and (ii) Al-doped HfO_2_ (Figure 1). The stacks were deposited on p-type Si (1 0 0) wafers. Prior to the deposition the substrates were chemically cleaned by using the standard Radio Corporation of America (RCA) process. HfO_2_ and Al_2_O_3_ were obtained using the following precursors: tetrakis(dimethylamido)hafnium (TDMAH) for HfO_2_ and trimethylaluminum (TMA) for Al_2_O_3_, while H_2_O vapor was employed for oxidation. The deposition temperature for the whole stack structure was T_dep_ = 135 °C. The stack composition for nanolaminated samples is a building block consisting of 20 cycles (cy) HfO_2_/over 5 cy Al_2_O_3_ repeated 5 times. Al-doped stacks were fabricated by 4 cy HfO_2_/1 cy Al_2_O_3_ block repeated 25 times. The overall thicknesses of both types of stacks were kept the same—16.5 nm, estimated by ellipsometric measurements of single HfO_2_ or Al_2_O_3_ layers grown on Si substrates. After deposition, half of the samples were subjected to rapid thermal annealing (RTA) in O_2_ at 800 °C for 1 min. Additionally, two single 20 nm reference layers of pure HfO_2_ and Al_2_O_3_ were deposited on Si substrate under the same ALD conditions, and half of them were RTA-treated.

It should be noticed that our previous investigations [17] revealed that RTA conducted under the above-mentioned conditions did not inflict any crystallization of the HfO_2_/Al_2_O_3_ stacks.

The X-ray photoelectron spectra were obtained using Al Kα (1486.6 eV) radiation in a VG ESCALAB MK II hemispherical electron spectrometer under a base pressure of 1 × 10^−8^ Pa. The spectrometer resolution was calculated from the Ag 3d_5/2_ line with the analyzer transmission energy of 20 eV. The full width at half maximum (FWHM) of this line was 1 eV. The spectrometer was calibrated against the Au 4f_7/2_ line (84.0 eV), and the sample charging was estimated from C 1s (285 eV) spectra from natural hydrocarbon contaminations on the surface. The measurement statistics (signal-to-noise ratio and energy step) allowed a binding energy (BE) accuracy of 0.2 eV to be achieved. The C 1s, O 1s, Al 2p, Si 2p, Hf 4f and Hf 4d core levels as well as the valence band (VB) regions were recorded at a normal take-off angle (90° to the surface). The take-off angle refers to the angle between the emitted photoelectrons (practical photoelectron detector position) and the sample surface. In the current study a take-off angle of 90° (normal emission) was used to ensure maximum sampling depth for maximum signal strength from thinner Al_2_O_3_ sublayers, which were placed under thicker HfO_2_ ones (see Figure 1). The photoelectron spectra were recorded and corrected by subtracting a Shirley-type background and quantified using the peak area and Scofield’s photoionization cross-sections [19]. For the fitting of the Hf 4f and O 1s peaks, a 30% Lorentz–Gauss peak shape was used. The O 1s peak was fitted with a single component, whereas the Hf 4f peak was fitted as a doublet with a spin–orbit splitting of 1.7 eV and an area ratio of 4:3. Peak fitting was performed using XPSPEAK4.1 software, where the resulting fitting error is given by the chi factor, which should ideally be equal to 1.

In order to elucidate possible relations between the chemical structure and the charge states in the investigated HfO_2_/Al_2_O_3_ stacks, MOS capacitors were prepared employing Al metallization. The Al-gate (top) electrode and Al-back contact to Si were deposited by evaporation with subsequent pattering of the gate by photolithography (gate area of 10^−4^ cm^2^). The charged defects present in the stacks were estimated through the high-frequency capacitance-voltage technique. The C-V characteristics were measured at room temperature using an Agilent E4890A LCR meter (Keysight, Santa Rosa, CA, USA) in a bias range from 2 V to −8 V at 1 MHz to ensure well pronounced saturation in inversion and accumulation regions. The voltage at which the measured capacitance equals the theoretical flat-band capacitance calculated using the Si doping level and the accumulation capacitance following [20] was designated as the flat-band voltage V_fb_.

## 3. Results

### 3.1. XPS Study

**Figure 2:**
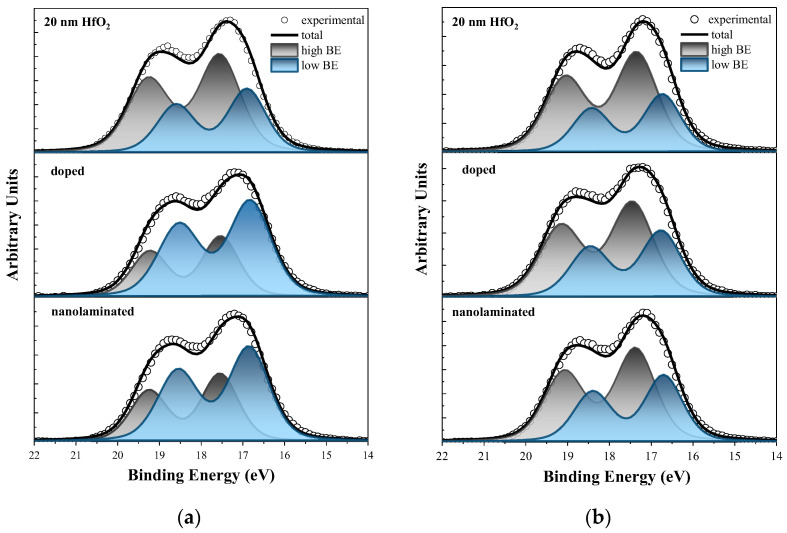
The Hf 4f XPS spectra deconvoluted into two Hf 4f doublets—low BE (blue) indicating the presence of Hf suboxides and high BE (gray) one associated with stoichiometric HfO_2_—(**a**) as-deposited and (**b**) after RTA.

XPS measurements were used to determine the chemical composition and bonding of as-deposited and annealed dielectric stacks. In Figure 2 the Hf 4f core level spectra for all samples (pure HfO_2_, laminated HfO_2_/Al_2_O_3_ and doped HfO_2_/Al_2_O_3_ stacks) before (Figure 2a) and after RTA (Figure 2b) are shown. Each Hf 4f spectrum was deconvoluted in two sets of double-peak components. Gaussian–Lorentzian line shapes were used for deconvolution of the spectra after standard Shirley background subtraction. The first doublet peaks are situated at BE of around 17.5 eV and 19.2 eV and correspond to the Hf_4+_ 4f_7/2_ and Hf_4+_ 4f_5/2_ energy levels of the stoichiometric HfO_2_ in which hafnium occurs in the 4+ oxidation state (Hf^4+^) [21]. The second doublet is shifted to lower BE and occurs at 16.9 eV and 18.6 eV, which corresponds to the 4f_7/2_ and 4f_5/2_ electronic states of Hf ^x+^ (x < 4) of the Hf suboxide [22,23]. The spin–orbit splitting of 1.7 eV is consistent with the usually reported value for the Hf 4f_7/2_ and Hf 4f_5/2_ doublet [24]. In Table 1 the positions of Hf_4+_ 4f_7/2_ and Hf_x+_ 4f_7/2_ energy levels and their FWHM for all stacks before and after annealing are summarized. No significant differences in the energy position of the stoichiometric and suboxide counterparts of the Hf 4f peak between the stacks are observed. After RTA, a small shift of 0.2 eV toward lower BE for the nanolaminated stack and pure HfO_2_ is observed. The FWHM also remains almost unchanged after RTA, revealing no increase in the disorder or changes in the amorphous/crystalline status of the films.

As seen in Figure 2a for the as-deposited pure HfO_2_ layer, the peak associated with the stoichiometric oxide bonds dominates the Hf 4f spectrum. On the contrary, for the as-deposited HfO_2_/Al_2_O_3_ stacks—both nanolaminated and doped ones—the low BE doublet dominates the Hf 4f spectra, i.e., Al inclusion increases Hf-suboxides (Figure 2a). The latter is in accordance with the results in [25] showing that Al-doping decreases the formation energy of oxygen vacancies caused by the Al substitution for Hf. Moreover, Al as a trivalent element is believed to form ionically compensated (2Al_Hf_)V_o_ and mixed compensated Al_Hf_V_o_ defects consisting of negatively charged Al ions and double positively charged oxygen vacancies. Interstitial Al could also lead to the formation of oxygen vacancies. The (2Al_Hf_)V_O_ defect has lower formation energy than the Al_Hf_V_O_ defect under both oxygen-rich and oxygen-poor conditions. Nevertheless, the Al_Hf_V_O_ defect might still form under oxygen-poor conditions since it does not require special placement of dopant atoms, unlike the (2Al_Hf_)V_O_ defect, which depends on a next-next-neighbor configuration of dopants and vacancy. The formation energy of an interstitial Al defect complex with oxygen vacancy is higher than that of (2Al_Hf_)V_O_ and Al_Hf_V_O_ [26]. After RTA, a significant increase in the Hf 4f peak corresponding to stoichiometric Hf^4+^ is observed in both nanolaminated and doped stacks, and its intensity is higher than that of the sub-stoichiometric one. Therefore, it can be assumed that RTA reduces the number of suboxides and improves the stoichiometry of HfO_2_/Al_2_O_3_ stacks. This is very well demonstrated in Table 1, where the area ratios of the Hf 4f peaks of stoichiometric and substoichiometric oxide components before and after RTA are also given. For doped stacks this ratio increases from 0.55 for the as-deposited to 1.53 for the annealed sample, i.e., by almost three times. As seen, there is also some improvement for the pure HfO_2_; however, the increase is insignificant (from about 1.7 to 1.9 after RTA).

**Figure 3:**
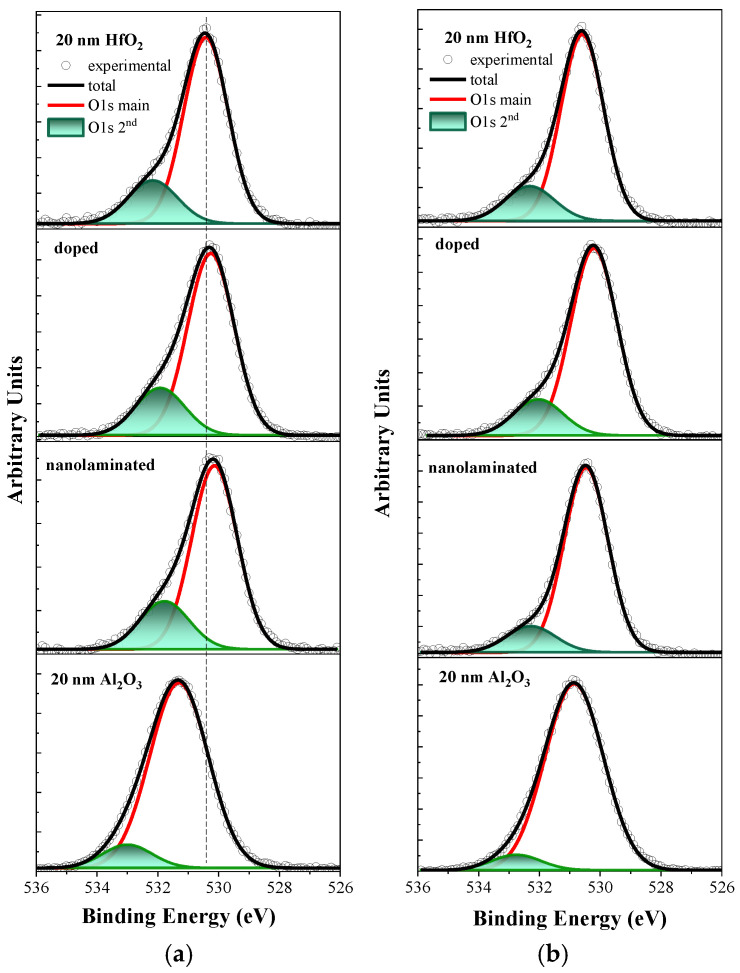
The O 1s core level deconvoluted into two contributions before (**a**) and after RTA in O_2_ (**b**).

In Figure 3 the O 1s spectra of pure HfO_2_, Al_2_O_3_ layers and HfO_2_/Al_2_O_3_ stacks are presented. For HfO_2_ and HfO_2_/Al_2_O_3_ stacks, O 1s spectra are fitted by two Gauss–Lorenzian peaks: the main one corresponding to Hf-O bonds at 530.1–530.4 eV and the second one at about 532 eV. The main O 1s contribution of the reference Al_2_O_3_ corresponding to Al-O bonds is positioned at higher BE of 531.30 eV, which is higher compared to the O 1s peak of the Hf-O bonds. The positions of the two O 1s peaks for all samples before and after RTA are given in Table 2. The O 1s peak position of the two HfO_2_/Al_2_O_3_ stacks is shifted by about 0.2–0.3 eV toward lower BE compared to the O 1s line of pure HfO_2_, which might be assigned to their sub-stoichiometric composition. The second O 1s peak located at 532.15 eV in pure HfO_2_ is usually associated with interstitial oxygen, oxygen vacancies, −OH bonds [24] or residual surface contaminants (e.g., C-O bonds) [27]. As seen (Table 2) this peak is shifted to lower BE for the two HfO_2_/Al_2_O_3_ stacks compared to pure HfO_2_. After RTA (Figure 3b, Table 2), the O 1s peak positions of pure HfO_2_ and doped stacks remain largely unaffected (a slight shift of about 0.1–0.2 eV to higher BE is observed). For the nanolaminated stack the shifts are slightly larger—0.3 eV to higher BE for the main O 1s peak shifts and 0.5 eV for the second O 1s peak.

In Table 2 the O 1s (main Me-O peak)/O 1s(second peak) ratio is also given. For the as-deposited pure HfO_2_ and Al_2_O_3_ films, this ratio is higher, which reflects their better stoichiometry and reduced defects. After RTA, the O 1s (main Me-O peak)/O 1s (second peak) ratio increases in all samples. The improved ratio can be explained with the reduced contribution from O vacancies, -OH bonds and C-O bonds after annealing. It is known that O_2_ RTA eliminates C, for example, in hafnium silicate (HfSiO) thin films [28].

**Figure 4:**
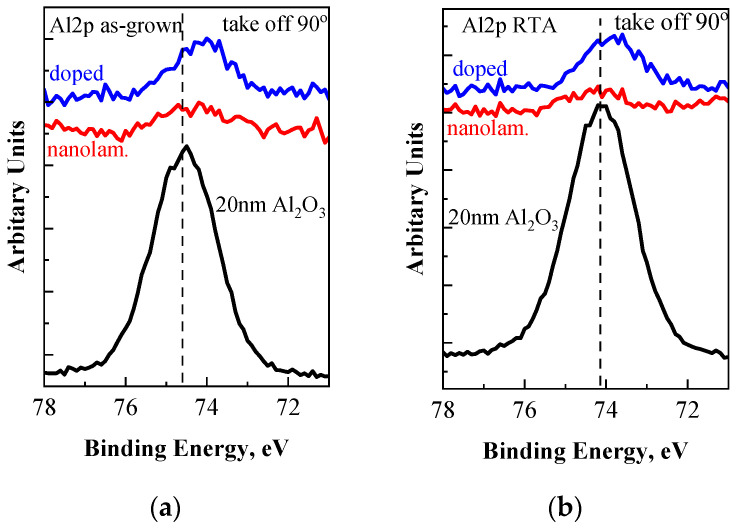
The Al 2p core level spectra of pure Al_2_O_3_ film and HfO_2_/Al_2_O_3_ stacks before (**a**) and after RTA (**b**).

The Al 2p core level spectra are shown in Figure 4. A single Gaussian line is used to fit the Al 2p peak as the spin–orbit splitting of Al 2p peak from Al_2_O_3_ is negligible. The Al 2p core level of both HfO_2_/Al_2_O_3_ stacks exhibits a shift of approximately 0.2–0.3 eV toward lower BE, indicating the formation of Hf–Al–O bonds [29,30]. This shift is better pronounced for the doped layers because of the larger number of HfO_2_/Al_2_O_3_ interfaces, which suggests a larger number of Hf-Al-O bonds. It should be noted, however, that the conjugated shift of the Hf 4f doublet with 0.2–0.3 eV toward higher BE compared to its position for “pure” HfO_2_ [29,31,32] is not observed. Considering the information depth of approximately 5 nm for the XPS method, the reduced intensity of the Al 2p peak in the nanolaminated stack can be readily explained by the position of the Al_2_O_3_ sublayer, located about 2.5 nm beneath the surface. After RTA, position of the Al 2p peak in the pure Al_2_O_3_ shifts by about 0.4 eV to lower BE. Several works have reported a similar shift of the Al 2p peak toward lower BE after thermal annealing [33,34]. Such a shift of the Al 2p peak at higher processing temperatures is mainly attributed to the decrease in the concentration of OH ions or coordination number of Al^3+^ ions in the film. The shift of the Al 2p peak in doped and laminated stacks after RTA is less pronounced, but its position remains at lower energies compared to that of the pure Al_2_O_3_.

**Figure 5:**
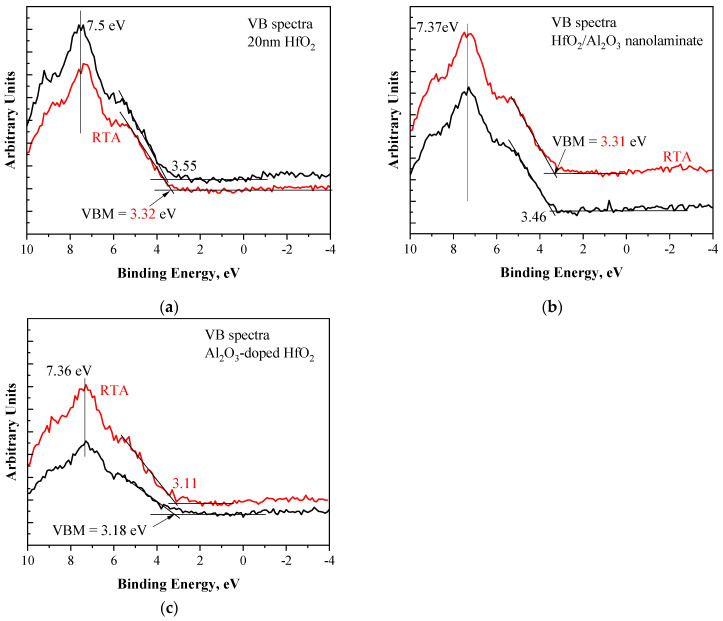
Valence band spectra showing the valence band maximum (VBM) values for pure HfO_2_ (**a**), nanolaminated (**b**), and doped (**c**) HfO_2_/Al_2_O_3_ stacks before and after RTA. The spectra are vertically shifted for visibility.

Valence band spectra were also investigated (Figure 5) to reveal the band alignments in different structures. To this end, the Hf 5d peak located at about 7.5 eV was measured. The valence band edge of oxide layer, *E_v_*(*ox*), was determined by linear extrapolation of the leading edge to the baseline of the valence band spectra. The valence band edge of dielectric layers before and after RTA are summarized in Table 3. Considering the valence band energy of Si, E_v_(Si), which is approximately 0.24 eV below the Fermi level (based on the substrate dopant concentration and the data in [35]), the valence band offset at the dielectric/Si interface, Δ*E_v_*, can be estimated as the difference between the valence band edges of the dielectric and of Si. A more precise determination of valence band offsets can be obtained by Kraut et al.’s equation [36]; however, this requires additional data. Nevertheless, the Δ*E_v_* values obtained here are in good agreement with reported data [37]. The largest Δ*E_v_*—3.31 eV—is observed for pure HfO_2_. Al introduction in the stacks results in a decrease in Δ*E_v_*, and this reduction is stronger for the doped stack. A noticeable reduction in Δ*E_v_* is observed after oxygen annealing for pure HfO_2_ and laminated stacks. For doped stacks, the reduction is smaller and could be considered within the experimental error. Therefore, both Al incorporation and RTA in O_2_ similarly influence the valence band offset at the dielectric/Si interface by decreasing its value.

### 3.2. Charges in the Stacks

**Figure 6:**
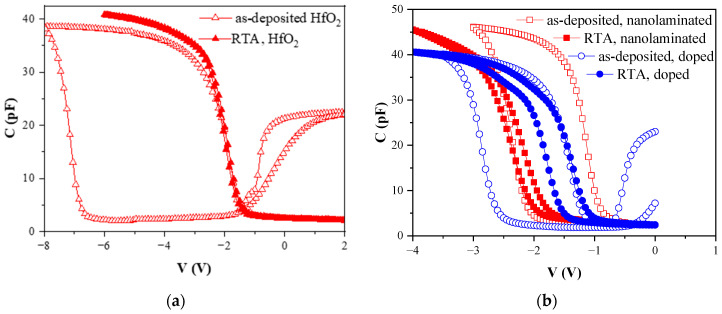
C-V curves of HfO_2_ (**a**) and laminated and doped HfO_2_/Al_2_O_3_ stacks (**b**) before and after RTA.

Electrically active defects in the stacks were investigated by measuring the C-V curves (Figure 6). The density of the initial oxide charge, denoted as Q_f_, is determined from the flatband voltage (V_fb_) extracted from the C-V curves. The flatband voltage corresponds to the applied voltage at which the capacitance equals the flatband capacitance. This capacitance is defined by the doping concentration of the Si and the capacitance of the dielectric stack. The relationship between V_fb_ and Q_f_ is given by [20]:

(1) $$V_{\mathrm{fb}}={\;\varphi }_{\mathrm{ms}}-Q_{f}/C_{0},$$

where C_0_ is the capacitance in accumulation and φ_ms_ is the metal–semiconductor work function difference. (The Al work function of 4.25 eV is used in calculations.) The value of Q_f_ is a net oxide charge and includes the charges in the high-k stack as well as the charges at the Si interface and the interfacial layer.

A hysteresis of the flat-band voltage V_fb_ when performing the C-V measurement from inversion to accumulation and back from accumulation to inversion is also detected. The C-V curves are nearly parallel to each other, revealing that the hysteresis of V_fb_ is mostly due to traps located within a tunneling distance from the interface that can easily communicate with the Si substrate by tunneling of electrons or holes and in this way changing their charge state and giving rise to the flat-band voltage shift. These traps are usually called “slow” or “border” traps [38], and their density can be approximately calculated by the following equation:

(2) $$Q_{\mathrm{sl}}=\Delta V_{\mathrm{fb}}C_{0},$$

where ΔV_fb_ is the hysteresis. It should be mentioned that the hysteresis gives only the lowest limit for the density of slow states [38]. Considering that a bias of up to −8 V was applied during the measurements to achieve accumulation saturation of the capacitance, at least part of the observed hysteresis could also be attributed to trapping/de-trapping processes in the bulk of the HfO_2_-based stacks.

The values of Q_f_, ΔV_fb_ and Q_sl_ for different stacks before and after RTA are given in Table 4. As seen, Q_f_ in HfO_2_ is positive, and its density is relatively high—3.5 × 10^12^ cm^−2^. Introduction of Al in HfO_2_ reduces Q_f_ significantly (by approximately three times) to 1.1–1.4 × 10^12^ cm^−2^. A similar strong reduction is also observed for the C-V hysteresis, which decreases from 3.2 V for the pure HfO_2_ to 1.2–1.5 V in the HfO_2_/Al_2_O_3_ stacks. The decrease in both Q_f_ and Q_sl_ is stronger for the nanolaminated stack. The large hysteresis of the C-V curves reveals that during the measurement, a large number of injected holes are trapped at the tunneling distance from the interface. The number of hole traps is significantly reduced in the Al-containing stacks. Therefore, it can be concluded that introduction of Al passivates defects in HfO_2_ and at the interface with Si, thus leading to a substantial decrease in Q_f_ and Q_sl_. A similar reduction in the hysteresis by Al introduction in HfO_2_ has also been observed by other authors [39]. Another explanation may be related to the compensation of the oxide charges associated with Al_2_O_3_ and HfO_2_ as the existence of negative oxide charge in thin Al_2_O_3_ films has been reported. In fact, the Al dopant in HfO_2_ (Hf substitution) is expected to act as a shallow acceptor center close (0.093 eV) to the valence band [40] occupied by an electron and hence is negatively charged. The Al substitution for Hf is expected to interact with neighbor oxygen vacancies and passivate them [40]. It should also be noted that the nature of oxygen vacancy in hafnia is quite complex; it could have multiple charge states, both negative and positive depending on the position of the Fermi level.

**Figure 7:**
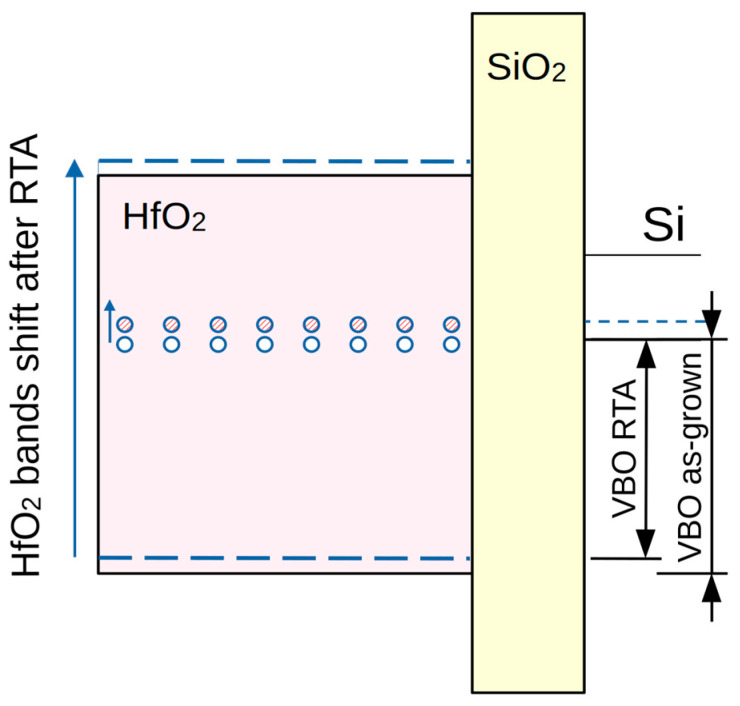
Schematic diagram of HfO_2_ bands shift upon annealing.

After RTA, Q_f_ remains mostly unaffected in the pure HfO_2_ and doped stacks, while it increases for the nanolaminated HfO_2_/Al_2_O_3_ stack, reaching the value of the pure HfO_2_. Taking into account the substantial decrease in suboxides in all stacks after RTA, it can be concluded that they are not the origin of the oxide charge. The hysteresis decreases substantially in all the layers, i.e., RTA in O_2_ reduces the density of slow traps. The reduction is most notable for pure HfO_2_, reaching one order of magnitude. This allows us to conclude that the origin of slow traps could be assigned to defects in HfO_2_. The increase in Q_f_ in nanolaminated stacks after RTA might be related to the change in the band diagram of the stacks and the reduction in the valence band offset at the dielectric/Si interface Δ*E_v_* (Table 3). The shift upward of *E_v_*(*ox*) with respect to *E_v_*(*Si*) changes the position of traps with respect to the Si valence band. Their energy position aligns with the Si bandgap; hence, they are inaccessible from the Si valence band. In this case, the donor type traps manifest as a fixed positive charge (Figure 7). This also explains the unchanged value of Q_f_ for the doped stack as its change in Δ*E_v_* is very small. The suggestion that the increased value of Q_f_ in nanolaminated stacks after RTA is due to the change in their energy position with respect to the Si valence band is supported also by the fact that the sum of Q_f_ and Q_sl_ before and after RTA is approximately equal, e.g., part of Q_sl_ converts into Q_f_ after RTA as a result of the change in the band gap alignment between the high-k stack and Si. This suggestion is supported by a recent paper [41] which investigates the energy position of different oxygen vacancy configurations in HfO_2_ and finds the energy position of one of these configurations to be about 2.02 eV below the conduction band of HfO_2_ almost aligned with the Si valence band.

An interesting question that arises from the presented data is on the origin of increased electron trapping in HfO_2_/Al_2_O_3_ stacks after RTA as reported in our previous investigations [16,17]. There are several factors that affect electron trapping at programming voltages (+V_g_) in the memory capacitors. The first is the density of electron traps. In HfO_2_, oxygen vacancies (most likely negatively charged ones, i.e., acting as acceptor levels [42] because they are situated in the upper half of HfO_2_ bandgap) are considered the main electron traps. Another alternative is the interstitial oxygen (which is related to the oxygen vacancies) that forms a deep acceptor level, but its position in the stable −2 charge state is 0.7 eV below the VB of Si [43]. The results here, however, suggest that the density of oxygen vacancies is reduced after the oxygen annealing. So, the more efficient electron trapping can be explained if we assume that the main trap sites are not the oxygen vacancies but Al-related defect states which increase/emerge after RTA, or alternatively the explanation could be derived from the effects as Coulombic repulsion. The Al-related trap sites are likely to form at HfO_2_/Al_2_O_3_ interfaces [11], especially after RTA. The smearing of HfO_2_/Al_2_O_3_ interfaces was clearly visible on TEM images after annealing [17]; however, this approach would lead to a clear effect of the number of HfO_2_/Al_2_O_3_ interfaces on the memory windows, which is not supported by our data [17]. The decrease in the C-V hysteresis upon annealing favors the repulsion hypothesis as high trap density close to the Si interface and subsequent electron trapping inevitably reduces the injection electric field and hence the tunneling current and the number of injected electrons in the stack. (The electrons trapped at and very near to the interface are lost after the programming voltage cut-off and do not contribute to stable memory window.) The second factor is the capture cross-section and energy level of the traps, and it may be suggested that annealing inflicts some changes in the nature of the traps such as close environment reconstruction that ease the trapping and produce high memory windows despite the decreased density. The third factor that could affect the number of trapped electrons is the level of the Fowler–Nordheim injection current. In the used capacitor configuration, i.e., p-type Si (corresponding to n-channel MOSFET), during the program voltage pulse, Si is in inversion. In this condition the current from the substrate to the stack is limited by the availability of the electrons (minority carriers) in the inversion layer, which in turn is maintained by thermal generation. Therefore, enhanced memory windows of RTA samples might be related to the increased generation rate due to changes in the Si surface region, which are not clear at the moment, but some results infer that such processes could take place [17].

## 4. Conclusions

The XPS study reveals that incorporation of Al into HfO_2_ results in an increased concentration of Hf-suboxides, which can be attributed to the reduced formation energy of oxygen vacancies induced by the substitution of Hf atoms with Al. The rapid thermal annealing in O_2_ enhances the stoichiometry of the HfO_2_/Al_2_O_3_ stacks and facilitates intermixing at the dielectric interface, resulting in the formation of Hf–Al–O bonds, as evidenced by the evolution of Al 2p core-level spectra. The incorporation of Al into HfO_2_ results in a pronounced reduction in both oxide charge (Q_f_) and slow trap charge (Q_sl_), which can be attributed to the passivation of defects within the HfO_2_ layer and at the HfO_2_/Si interface. This defect passivation also leads to a substantial decrease in the number of hole traps in the Al-containing stacks. The obtained results imply that suboxides are not the primary source of oxide charge. Moreover, RTA in O_2_ effectively decreases the density of slow traps, with the most pronounced effect observed for pure HfO_2_, where a reduction of nearly one order of magnitude is achieved. This finding supports the assignment of slow traps to defects intrinsic to HfO_2_. The reduction in the valence band offset (ΔE_v_) between the dielectric and the Si substrate inflicted by both the Al incorporation and the post-deposition RTA in O_2_ may also affect the electrical behavior of traps. The enhanced electron trapping observed in O_2_-annealed stacks can be rationalized by assuming that the dominant trap states are not oxygen vacancies but rather Al-related defect states, which either increase in density or become activated during RTA.

## Figures and Tables

**Table 1:** Energy position of Hf 4f_7/2_ peak of stoichiometric and suboxide counterparts, their FWHM and the area ratio of the Hf 4f peaks of stoichiometric and substoichiometric oxide components before and after RTA.

Sample	Hf 4f Peak	Hf 4f_7/2_ Position, eV		FWHM, eV		Hf 4f (Stoichiometric)/Hf 4f (Suboxide) Peak Area Ratio	
		As-Grown	RTA	As-Grown	RTA	As-Grown	RTA
pure HfO_2_	Stoichiometric	17.6	17.4	1.2	1.2	1.69	1.92
	Suboxide	16.9	16.7	1.1	1.1		
doped	Stoichiometric	17.5	17.5	1.0	1.2	0.55	1.53
	Suboxide	16.8	16.8	1.2	1.1		
nanolaminated	Stoichiometric	17.6	17.4	1.1	1.1	0.66	1.5
	Suboxide	16.9	16.7	1.1	1.1		

**Table 2:** Energy positions of the main Me-O O 1s peak and the second O 1s as well as the O 1s (main)/O 1s (2nd) area ratio before and after RTA.

Sample	Peak	O 1s Position, eV		O 1s (Main)/O 1s (2nd) Area Ratio	
		As-Deposited	RTA	As-Deposited	RTA
pure HfO_2_	main Me-O peak	530.45	530.6	3.9	4.7
	second peak	532.15	532.3		
doped	main Me-O peak	530.3	530.2	3.6	4.9
	second peak	531.9	532.0		
nanolaminated	main Me-O peak	530.1	530.4	3.5	6.3
	second peak	531.8	532.3		
pure Al_2_O_3_	main Me-O peak	531.30	530.8	9.4	14.4
	second peak	533.00	532.75		

**Table 3:** Valence band edges of dielectric E_v_(ox) and valence band offsets between dielectrics and Si for different stacks before and after RTA.

Sample	Valence Band Edge, *E_v_*(ox) eV	Valence Band Offset Δ*E_v_*, eV
nanoalminated, as-grown	3.46	3.22
nanolaminated, RTA	3.31	3.07
doped, as-grown	3.18	2.94
doped, RTA	3.11	2.87
HfO_2_, as-grown	3.55	3.31
HfO_2_, RTA	3.32	3.08

**Table 4:** The values of fixed oxide charge, flat-band hysteresis and border traps for different stacks.

Sample	Treatment	Q_f_, cm^−2^	Hysteresis V_fb_, V	Q_sl_, cm^−2^
Pure HfO_2_	as-grown	3.5 × 10^12^	3.2	8.9 × 10^12^
	RTA	3.5 × 10^12^	0.3	8.8 × 10^11^
Nanolaminated	as-grown	1.1 × 10^12^	1.2	3.5 × 10^12^
	data	3.9 × 10^12^	0.3	1.1 × 10^12^
Doped	as-grown	1.4 × 10^12^	1.5	4.3 × 10^12^
	RTA	1.2 × 10^12^	0.8	2 × 10^12^

## Data Availability

The original contributions presented in this study are included in the article. Further inquiries can be directed to the corresponding author.

## Abbreviations

The following abbreviations are used in this manuscript:

CTM	Charge-Trapping Memory
CTL	Charge-Trapping Layer
RCA	Radio Corporation of America
RTA	Rapid Thermal Annealing
BE	Binding Energy
FWHM	Full Width at Half Maximum
VB	Valence Band
VBO	Valence Band Offset
C-V	Capacitance–Voltage
MOS	Metal Oxide Semiconductor
